# The implication of lncRNA expression pattern and potential function of lncRNA RP4‐576H24.2 in acute myeloid leukemia

**DOI:** 10.1002/cam4.2518

**Published:** 2019-09-30

**Authors:** Jifu Zheng, Yuan Song, Zhenjiang Li, Aiping Tang, Yan Fei, Wenfeng He

**Affiliations:** ^1^ Key Laboratory of Hematology of Jiangxi Province Department of Hematology The Second Affiliated Hospital of Nanchang University Nanchang China; ^2^ Key Laboratory of Molecular Medicine of Jiangxi The Second Affiliated Hospital of Nanchang University Nanchang China

**Keywords:** acute myeloid leukemia, disease risk, lncRNA RP4‐576H24.2, long noncoding RNA expression pattern, prognosis

## Abstract

**Background:**

Recent studies have revealed that long noncoding RNAs (lncRNAs) may hold crucial triggers of the pathogenesis of hematological malignancies, while the studies evaluating the expression pattern of lncRNA in acute myeloid leukemia (AML) are few. Thus, this study aimed to investigate the implication of lncRNA expression pattern in AML development and progression.

**Methods:**

Bone marrow samples from four AML patients and four controls were subjected to lncRNA sequencing. Then, bone marrow samples from 110 AML patients and 40 controls were proposed to real‐time quantitative polymerase chain reaction (RT‐qPCR) validation for 10 candidate lncRNAs. Clinical data and survival profiles were recorded in AML patients. Furthermore, lncRNA RP4‐576H24.2 expression in AML cell lines and its effect on AML cell proliferation and apoptosis were detected.

**Results:**

LncRNA expression pattern by sequencing clearly distinguished AML patients from controls, and 630 upregulated and 621 downregulated lncRNAs were identified in AML patients compared to controls, which were mainly enriched in AML oncogene‐related biological process and pathways (such as neutrophil degranulation, leukocyte transendothelial migration, and hematopoietic cell lineage). RT‐qPCR validation observed that six lncRNAs correlated with AML risk, one lncRNA associated with risk stratification, and three lncRNAs correlated with survivals, among which lncRNA RP4‐576H24.2 was the only one correlated with AML susceptibility, risk stratification, and survivals. Further in vitro experiments showed that lncRNA RP4‐576H24.2 was upregulated in AML cell lines compared to normal bone marrow mononuclear cells (BMMCs), and promoted proliferation while inhibited apoptosis in HL‐60 and KG‐1 cells.

**Conclusions:**

LncRNA expression pattern is closely involved in the development and progression of AML, and several specific lncRNAs exhibit potential to be biomarkers for AML risk and prognosis. Besides, lncRNA RP4‐576H24.2 might be a potential oncogene in AML pathogenesis.

## Graphical abstract

This study aimed to investigate the implication of long noncoding RNA (lncRNA) expression pattern in acute myeloid leukemia (AML) pathogenesis. It showed that lncRNA expression pattern is closely involved in the development and progression of AML, and several specific lncRNAs exhibit potential to be biomarkers for AML risk and prognosis. Besides, lncRNA RP4‐576H24.2 is discovered to be a key oncogene in AML pathogenesis.

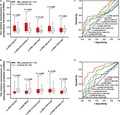

## 1 INTRODUCTION

Acute myeloid leukemia (AML), a complex hematological malignancy characterized by its heterogenetic cytology, presents poor long‐term outcome in the majority of adult patients.^1^ Although AML patients’ prognosis has improved, the proportion of patients achieving long‐term survival is still insufficient referring to that only 40% in younger adult and 10% in the elderly obtain long‐term survival.^1^, ^2^ Therefore, management of AML patients pivots around enhancing recovery and survival, in which discovering assistant prognostic factors to enhance the management of AML patients plays a relatively crucial role. Besides the chromosomal dysregulations and gene mutations that have been used in risk stratification, several other genetic factors also present with potentials in AML risk and prognosis prediction, which include diversified kinds of noncoding ribonucleic acid (ncRNA).^3^, ^4^

Long noncoding RNA (lncRNA) is a class of RNAs with more than 200 nucleotides which have less protein‐coding capacities, those molecules function as key regulators in cellular activities (such as the process of cell cycle, differentiation, and imprinting).^5^, ^6^, ^7^, ^8^, ^9^ Recent studies have revealed that lncRNAs may hold crucial triggers of the development and progression of hematological malignancies.^10^ In AML, the studies aiming at investigating the role of lncRNA are limited, while the findings are minimal yet intriguing. Previous studies reveal that some specific lncRNAs might play crucial roles in regulating the AML cell functions or chemoresistance, for instance, a previous study reports that lncRNA urothelial carcinoma‐associated 1 (UCA1) knockdown inhibits chemoresistance via repressing glycolysis by mediating the microRNA (miR)‐125a/hexokinase 2 signaling pathway.^11^, ^12^ However, the existed studies mostly focus on investigating the roles of several specific/individual lncRNAs in AML, such as lncRNA antisense noncoding RNA in the INK4 locus (ANRIL), lncRNA UCA1 and so on, while the studies assessing the lncRNA expression pattern in AML are few.^11^, ^13^

Herein, we conducted this study to investigate the implication of lncRNA expression pattern in AML pathogenesis and the potential of several specific candidate lncRNAs as markers for AML risk and prognosis, and further explore the effect of lncRNA RP4‐576H24.2 on regulating AML progression.

## 2 METHODS

### 2.1 Patients and controls

About 110 de novo AML patients between July 1, 2015 and June 31, 2018 were consecutively enrolled in our study. The inclusion criteria were: (a) Diagnosed as de novo AML patients according to the World Health Organization (WHO) Classification of Tumors of Hematopoietic and Lymphoid Tissues (2008); (b) Age above 18 years. The exclusion criteria were: (a) Secondary or mixed AML; (b) History of other solid tumor or hematological malignancies; (c) Previous treatment with chemotherapy or radiotherapy. Meanwhile, 40 participants who were adult bone marrow donors or adult patients with nonmalignancy diseases (such as thrombocytopenic purpura and myelofibrosis) who underwent bone marrow biopsy were enrolled as controls. This present study was approved by the Ethics Committee of our hospital, all participants (AML patients and controls) provided written informed consents.

### 2.2 Data collection, sample acquisition, treatment, and assessment

Age, gender, French‐American‐British (FAB) classification, white blood cell (WBC), cytogenetics, molecular genetics, and risk stratification (according to National Comprehensive Cancer Network [NCCN] Clinical Practice Guidelines in Oncology of AML [Version 2, 2013]) of AML patients were documented. And, bone marrow samples were obtained from AML patients during biopsy. Patients were treated in accordance with NCCN Clinical Practice Guidelines in Oncology of AML (Version 2, 2013). Treatment remission status of induction chemotherapy was assessed, and complete remission (CR) was defined as bone marrow with at least 20% cellularity and BM blasts below 5% at steady state after treatment, without cytological evidence of leukemia, no transfusion requirement, leukocyte count above 1 × 10^9^/L, and platelet count above 100 × 10^9^/L. Patients were then followed up, the last follow‐up date was June 31, 2018 and the median follow‐up duration was 17.0 (range 1.0‐36.0) months, and event‐free survival (EFS) as well as overall survival (OS) were calculated. The EFS was defined as the time interval from initiation of treatment to disease recurrence, progression, or death, and the OS was defined as the time interval from initiation of treatment to death. Besides, bone marrow samples were also acquired from controls by biopsy as well.

### 2.3 RNA sequencing process

Four de novo AML patients and four age‐ and gender‐matched controls were randomly selected from total participants, and their bone marrow samples were subjected to RNA sequencing by Genergy Bio Company. In brief, (a) Total RNA was extracted from bone marrow using PureZOL RNA isolation reagent (Bio‐Rad), and then concentration, purity, and integrity were assessed and adjusted; (b) Ribosomal RNA (rRNA) was removed by Epicentre Ribo‐zero™ rRNA Removal Kit (Epicentre) and the remaining RNA was proposed to generate sequencing library according to the methods in a previous study^14^; (c) The library was then sequenced on Illumina Hiseq X10 platform (Illumina); (d) Trimmed reads were mapped to the human genome Hg38 by HISAT2 with the default parameters; (e) The gene (lncRNAs and mRNAs) read count was calculated using FeatureCounts.

### 2.4 Bioinformatics

The following bioinformatic analyses were carried out based on the sequencing data of lncRNA and mRNA expression patterns: (a) Principal component analysis (PCA) of gene expression pattern (lncRNA and mRNA individually) was performed by Stats package; (b) Heatmap analysis of gene expression pattern (lncRNA and mRNA individually) was performed by Pheatmap package; (c) Dysregulated genes (lncRNA and mRNA individually) were analyzed by DeSeq2 package and showed as volcano spot with the statistical significance defined as *P* value <.05 and the biological significance defined as a difference of fold change (FC) above 2.0 times; (d) Heatmap analysis of dysregulated genes (lncRNA and mRNA individually) was performed by Pheatmap package; (e) Gene Ontology (GO) and Kyoto Encyclopedia of Genes and Genomes (KEGG) enrichment analysis of dysregulated lncRNAs were performed using DAVID web server according to their regulated mRNAs, afterward, GO and KEGG enrichment analysis of dysregulated mRNAs were also performed; (f) Regulatory network of top 40 dysregulated lncRNAs (20 upregulated and 20 downregulated) was drawn by Igraph package based on their regulated mRNAs; (g) Circos graph for transcription and regulation information was drawn by RCircos package. R Software (Version 3.5.3) (Lucent Technologies) was used for bioinformatic analysis. The genes (lncRNAs and mRNAs) which were identified in 50% or above samples were included in the bioinformatics analysis.

### 2.5 Measurement of 10 candidate lncRNAs by real‐time quantitative polymerase chain reaction (RT‐qPCR) validation

So as to validate the correlation of some potential lncRNA expressions with disease susceptibility, risk stratification, and prognosis, 10 candidate lncRNAs were chosen according to their absolute value of Log_2_FC, which included top 5 upregulated and top 5 downregulated lncRNAs in AML patients compared to controls in RNA sequencing as follows: lncRNA CES1P1, lncRNA RP4‐576H24.2, lncRNA LINC01262, lncRNA SIGLEC16, lncRNA OR7E140P, lncRNA DIO3OS, lncRNA MEG3, lncRNA ST3GAL6‐AS1, lncRNA OR51A10P, and lncRNA RP5‐983L19.2. Then, their expressions in bone marrow samples from 110 de novo AML patients and 40 controls were validated by RT‐qPCR.

### 2.6 In vitro experiments

Human AML cell lines HL‐60, HT‐93, KG‐1, and AML‐193 were purchased from Leibniz Institute DSMZ‐German Collection of Microorganisms and Cell Cultures. HL‐60, HT‐93, and KG‐1 cells were cultured in 90% Roswell Park Memorial Institute (RPMI) 1640 Medium (Gibco) and 10% fetal bovine serum (Gibco), AML‐193 cells were cultured in 90% Iscove's Modified Dulbecco's Medium (Gibco) and 10% fetal bovine serum (Gibco). Besides, human normal bone marrow mononuclear cells (BMMCs) were isolated from a healthy donor as controls. Then, the relative expression of lncRNA RP4‐576H24.2 was detected in HL‐60, HT‐93, KG‐1, AML‐193 cell lines and controls. Afterward, control overexpression, lncRNA RP4‐576H24.2 overexpression, control shRNA, and lncRNA RP4‐576H24.2 shRNA plasmids were constructed by Shanghai QeeJen Bio‐Tech Company and transfected into HL‐60 cells as well as KG‐1 cells, which were divided into NC(+), Lnc(+), NC(−), and Lnc(−) groups. Subsequently, lncRNA RP4‐576H24.2 relative expression in each group was detected by RT‐qPCR at 24 hours posttransfection; cell proliferation in each group was detected by Cell Counting Kit ‐ 8 (Sigma) at 0, 24, 48, and 72 hours posttransfection; finally, the cell apoptosis rate in each group was detected by Annexin V Apoptosis Detection Kit FITC (Thermo) at 24 hours posttransfection. All the cell experiments were performed in triplicate.

### 2.7 RT‐qPCR

First of all, the total RNA was isolated from bone marrow samples or cells using PureZOL RNA isolation reagent (Bio‐Rad) and was evaluated by a spectrophotometer. Then, the total RNA was reversely transcribed into complementary DNA using the QuantiNova Reverse Transcription Kit (Qiagen). Subsequently, PCR was performed by QuantiNova SYBR Green PCR Kit (Qiagen) and the results were calculated with 2^−△△Ct^ formula using U6 or GADPH as an internal reference. And, the primers used in RT‐qPCR are listed in Table 1.

**Table 1:** Primers list

Gene	Forward Primer (5′‐3′)	Reverse Primer (5′‐3′)
LncRNA CES1P1	TAGAATCACTGAGGCACCAATG	AGAGACACAAGACACCATCACT
LncRNA RP4‐576H24.2	GCTGATGCTGGCACCTATTACT	GCGACTGACACGGACTTCTC
LncRNA LINC01262	CAACAAGCCGTCACTGGAACT	TGGCGGAGATAGCACTGGTAA
LncRNA SIGLEC16	AGAGCCCAGAGATGCTGCT	CAAGACACGATGACACACAGG
LncRNA OR7E140P	CAGAGCCACGGAATCTCACAG	CAGCACAGGTTGGAGAGGAAG
LncRNA DIO3OS	GCCTTCCTGCTCTTCGTTGT	CGCTACCTGCTCTGAGATGTG
LncRNA MEG3	GGATGAGGAAGGAGGCTGTG	GGAATACGGTGGTCTGGTGAA
LncRNA ST3GAL6‐AS1	GTGGCTTCAGGACAAGGACTT	CACATCTTCAGGCATCACATCC
LncRNA OR51A10P	CACTGCTCATCCTCCTCTCCTA	TGTAGTCTCCTGCGAATCTCCT
LncRNA RP5‐983L19.2	CTCTTGACAATGCCTGCTCCT	CATCCTCGCTAACACGGTGAA
GAPDH	GAGTCCACTGGCGTCTTCAC	ATCTTGAGGCTGTTGTCATACTTCT

### 2.8 Statistical analysis

Statistical analysis was performed by SPSS Software 21.0 (IBM), while the statistical image was drawn by GraphPad Prism Software 7.01 (GraphPad Int, co, Ltd.). Comparison among multiple groups was detected by one‐way analysis of variance (ANOVA) followed by multiple comparison test or Kruskal‐Wallis H test. Comparison between two groups was detected by *t* test or Wilcoxon rank sum test. Predictive value of candidate parameters for disease risk was detected by receiver operating characteristic (ROC) curve and assessed by area under the curve (AUC). Comparison of EFS and OS was detected by Kaplan‐Meier curve and log‐rank test. *P* value <.05 was considered as significant.

## 3 RESULTS

### 3.1 Characteristics of AML patients in RNA sequencing

A total of four AML patients and four controls were randomly selected and included in RNA sequencing. For AML patients included in RNA sequencing, the mean age was 42.5 ± 10.3 years, and the numbers of females as well as males were both 2 (50.0%) (Table 2). In addition, the numbers of patients with FAB classification M1, M2, M4, M5, and M6 were 0 (0.0%), 2 (50.0%), 1 (25.0%), 1 (25.0%), and 0 (0.0%), respectively. There were 2 (50.0%), 1 (25.0%), and 1 (25.0%) patients who had risk stratification of better‐risk, intermediate‐risk, and poor‐risk, and the median value of WBC was 42.1 (11.7‐81.8) × 10^9^/L. Other detailed information of the four AML patients’ characteristics is shown in Table 2.

**Table 2:** AML patients’ characteristics

Parameters	RNA sequencing (N = 4)	RT‐qPCR validation (N = 110)
Age (years), mean ± SD	42.5 ± 10.3	43.1 ± 13.4
Gender, No. (%)		
Male	2 (50.0)	63 (57.3)
Female	2 (50.0)	47 (42.7)
FAB classification, No. (%)		
M1	0 (0.0)	3 (2.7)
M2	2 (50.0)	35 (31.8)
M4	1 (25.0)	31 (28.2)
M5	1 (25.0)	34 (30.9)
M6	0 (0.0)	7 (6.4)
Cytogenetics, No. (%)		
−5 or −5q	0 (0.0)	1 (0.9)
inv(3) or t(3;3)	0 (0.0)	1 (0.9)
t(9;22)	0 (0.0)	2 (1.8)
11q23	0 (0.0)	3 (2.7)
t(9;11)	0 (0.0)	3 (2.7)
−7 or 7q‐	0 (0.0)	4 (3.6)
+8	0 (0.0)	4 (3.6)
t(8;21)	1 (25.0)	7 (6.4)
inv(16) or t(16;16)	0 (0.0)	9 (8.2)
Others	0 (0.0)	11 (10.0)
CK	1 (25.0)	11 (10.0)
NK	2 (50.0)	54 (49.1)
Monosomal karyotype, No. (%)	0 (0.0)	7 (6.4)
FLT3‐ITD mutation, No. (%)	0 (0.0)	25 (22.7)
Isolated biallelic CEBPA mutation, No. (%)	0 (0.0)	8 (7.3)
NPM1 mutation, No. (%)	1 (25.0)	31 (28.2)
Risk stratification, No. (%)		
Better‐risk	2 (50.0)	28 (25.5)
Intermediate‐risk	1 (25.0)	45 (40.9)
Poor‐risk	1 (25.0)	37 (33.6)
WBC (×10^9^/L), median (IQR)	42.1 (11.7‐81.8)	15.4 (6.9‐28.4)

Abbreviations: AML, acute myeloid leukemia; CEBPA, CCAAT/enhancer‐binding protein α; CK, complex karyotype; FAB classification, French‐American‐Britain classification systems; FLT3‐ITD, internal tandem duplications in the FMS‐like tyrosine kinase 3; IQR, interquartile range; NK, normal karyotype; NPM1, nucleophosmin 1; RT‐qPCR, real‐time quantitative polymerase chain reaction; SD, standard deviation; WBC, white blood cell.

### 3.2 LncRNA and mRNA expression patterns in AML patients

**Figure 1:**
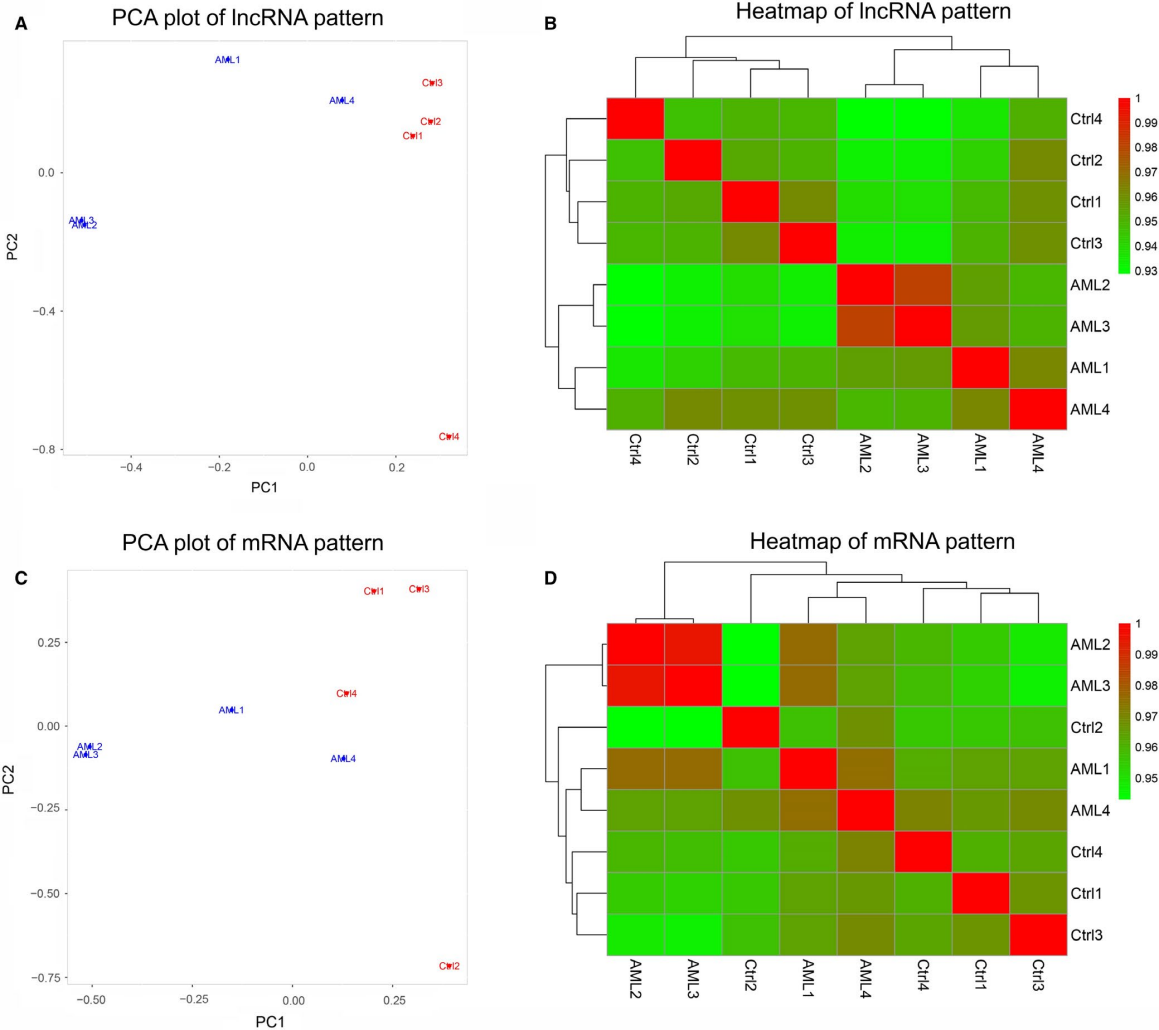
LncRNA and mRNA expression patterns. PCA plot of lncRNA expression pattern (A), heatmap of lncRNA expression pattern (B), PCA plot of mRNA expression patterns (C), and heatmap of mRNA expression pattern (D) between four AML patients and four controls. PCA, principal component analysis; AML, acute myeloid leukemia; lncRNA, long noncoding RNA

**Figure 2:**
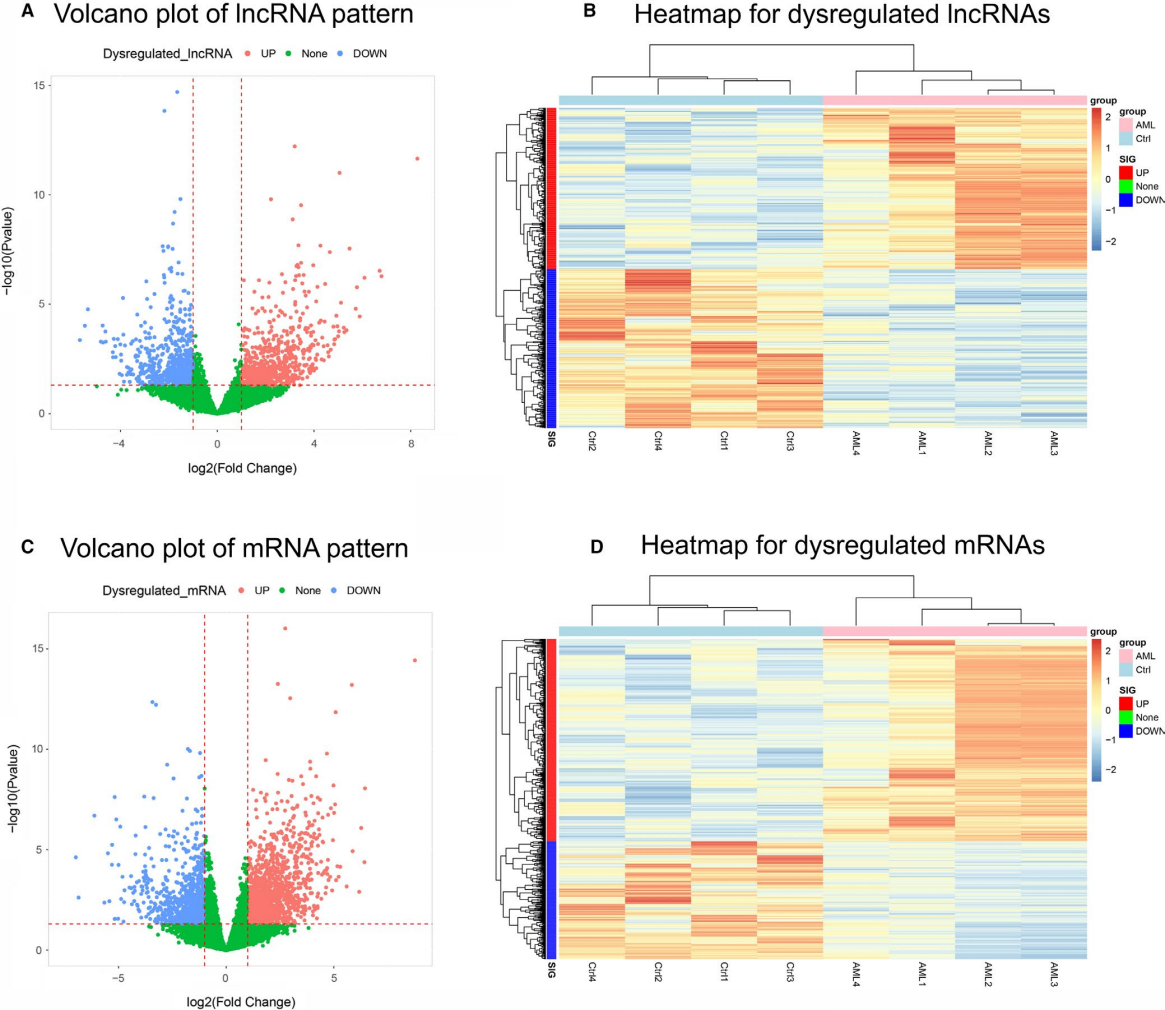
Dysregulated lncRNAs and mRNAs. Upregulated lncRNAs (red dots), downregulated lncRNAs (blue dots), and unchanged lncRNAs (green dots) in volcano plot (A), heatmap of dysregulated lncRNAs (B), upregulated mRNAs (red dots), downregulated mRNAs (blue dots), and unchanged mRNAs (green dots) in volcano plot (C) and heatmap of dysregulated mRNAs (D) between four AML patients and four controls. AML, acute myeloid leukemia; lncRNA, long noncoding RNA

PCA plot analysis disclosed that lncRNA expression pattern could differentiate AML patients from controls (Figure 1A), and heatmap also showed that lncRNA expression pattern distinguished AML patients from controls with a good intra‐group correlation in both AML patients and controls (Figure 1B). Furthermore, PCA plot disclosed that AML patients and controls could be distinguished by the mRNA patterns as well (Figure 1C), and heatmap also displayed that mRNA patterns could differentiate AML patients from controls with good intro‐group correlation in both groups (Figure 1D). Then the volcano plot identified 630 upregulated lncRNAs and 621 downregulated lncRNAs in AML patients compared with controls (Figure 2A), and heatmap for dysregulated lncRNAs displayed stratifying intra‐group correlation in both AML patients and controls (Figure 2B). Similarly, volcano plot identified 1357 upregulated mRNAs and 790 downregulated mRNAs in AML patients than those in controls (Figure 2C), and heatmap of dysregulated mRNAs also displayed that the intro‐group correlation was good in both AML patients and controls (Figure 2D). These results indicated that the lncRNA expression profile is implicated in the development of AML.

### 3.3 Enrichment analysis of dysregulated lncRNAs/mRNAs

**Figure 3:**
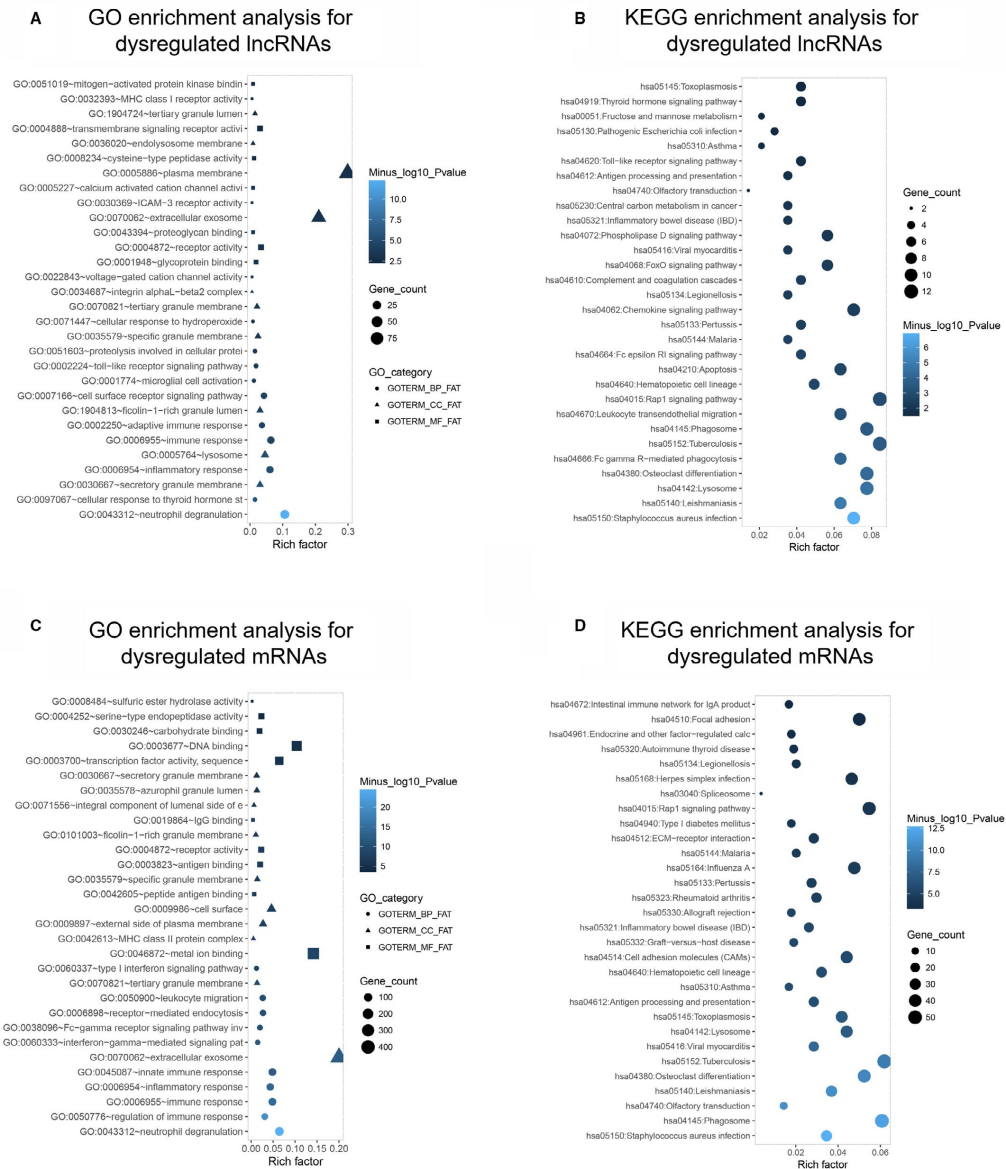
Enrichment analysis of dysregulated lncRNAs and mRNAs. GO enrichment analysis of dysregulated lncRNAs (A), KEGG enrichment analysis of dysregulated lncRNAs (B), GO enrichment analysis of dysregulated mRNAs (C), KEGG enrichment analysis of dysregulated mRNAs (D) between four AML patients and four controls. GO, Gene Ontology; KEGG, Kyoto Encyclopedia of Genes and Genomes; AML, acute myeloid leukemia; lncRNA, long noncoding RNA

Then, for the purpose of evaluating the potential regulatory role of dysregulated lncRNAs and mRNAs in AML pathogenesis, enrichment analysis was performed, and the GO enrichment analysis revealed that the dysregulated lncRNAs were enriched in biological processes (such as neutrophil degranulation, inflammatory response, and immune response), cellular component (including secretory granule membrane, lysosome, and ficolin‐1‐rich granule lumen), and molecular functions (including voltage‐gated cation channel activity, glycoprotein binding, and proteoglycan binding) (Figure 3A), and the KEGG analysis showed that the dysregulated lncRNAs were mainly correlated with pathways related to AML pathogenesis including leukocyte transendothelial migration pathways, hematopoietic cell lineage pathways, and apoptosis pathways (Figure 3B). As for the dysregulated mRNAs, GO enrichment analysis presented that they were mainly associated with biological processes (such as neutrophil degranulation, regulation of immune response, and inflammatory response), cellular component (such as extracellular exosome, external side of plasma membrane, and cell surface), and molecular functions (such as metal ion binding, peptide antigen binding, and antigen binding) (Figure 3C), and the KEGG enrichment analysis disclosed that the dysregulated mRNAs were enriched in several pathways that were correlated with AML pathogenesis, such as hematopoietic cell lineage, graft‐versus‐host disease, and allograft rejection (Figure 3D).

### 3.4 Regulatory network of top 40 dysregulated lncRNAs

**Figure 4:**
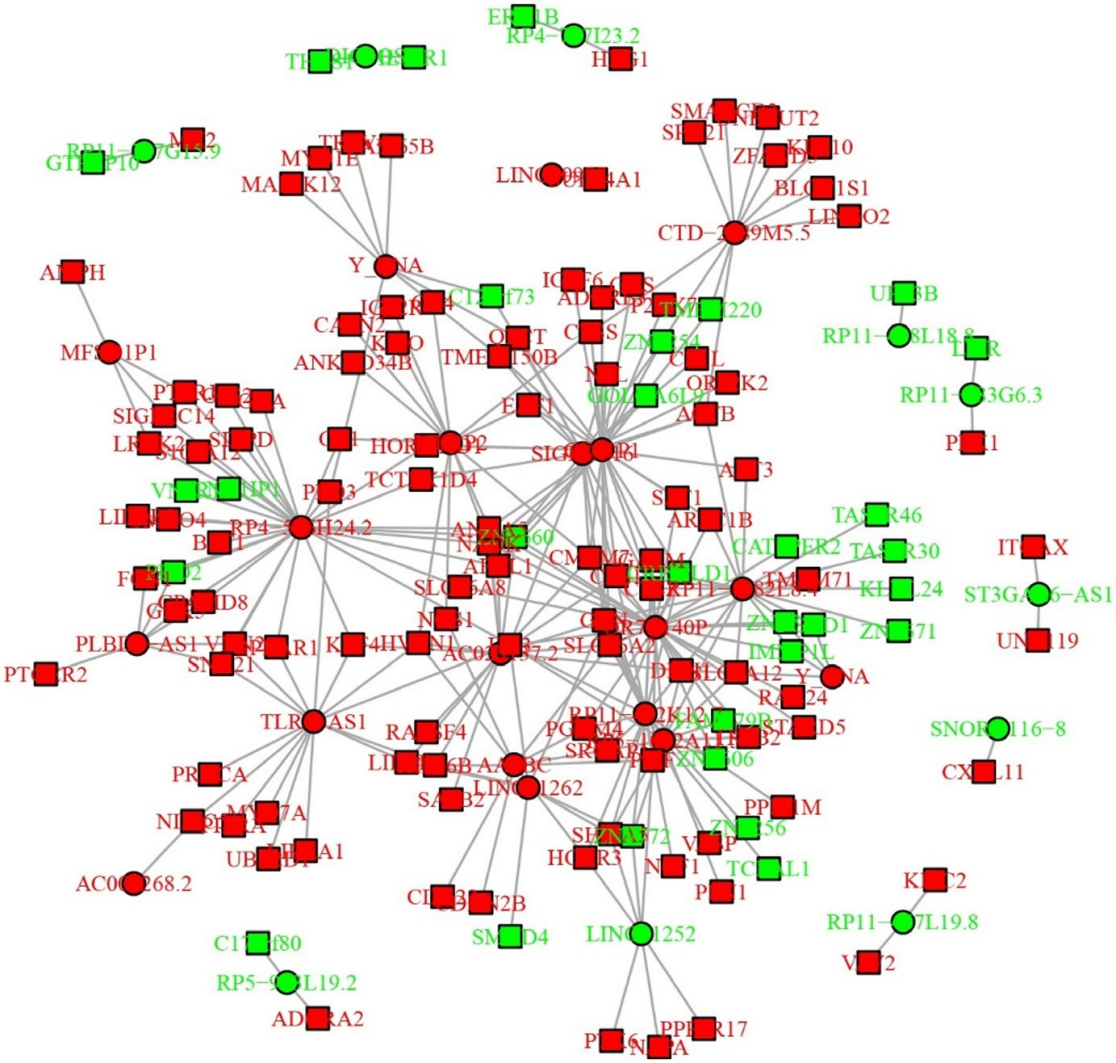
Regulatory network. The interactions of top 20 upregulated lncRNAs and top 20 downregulated lncRNAs with mRNAs. lncRNA, long noncoding RNA

**Figure 5:**
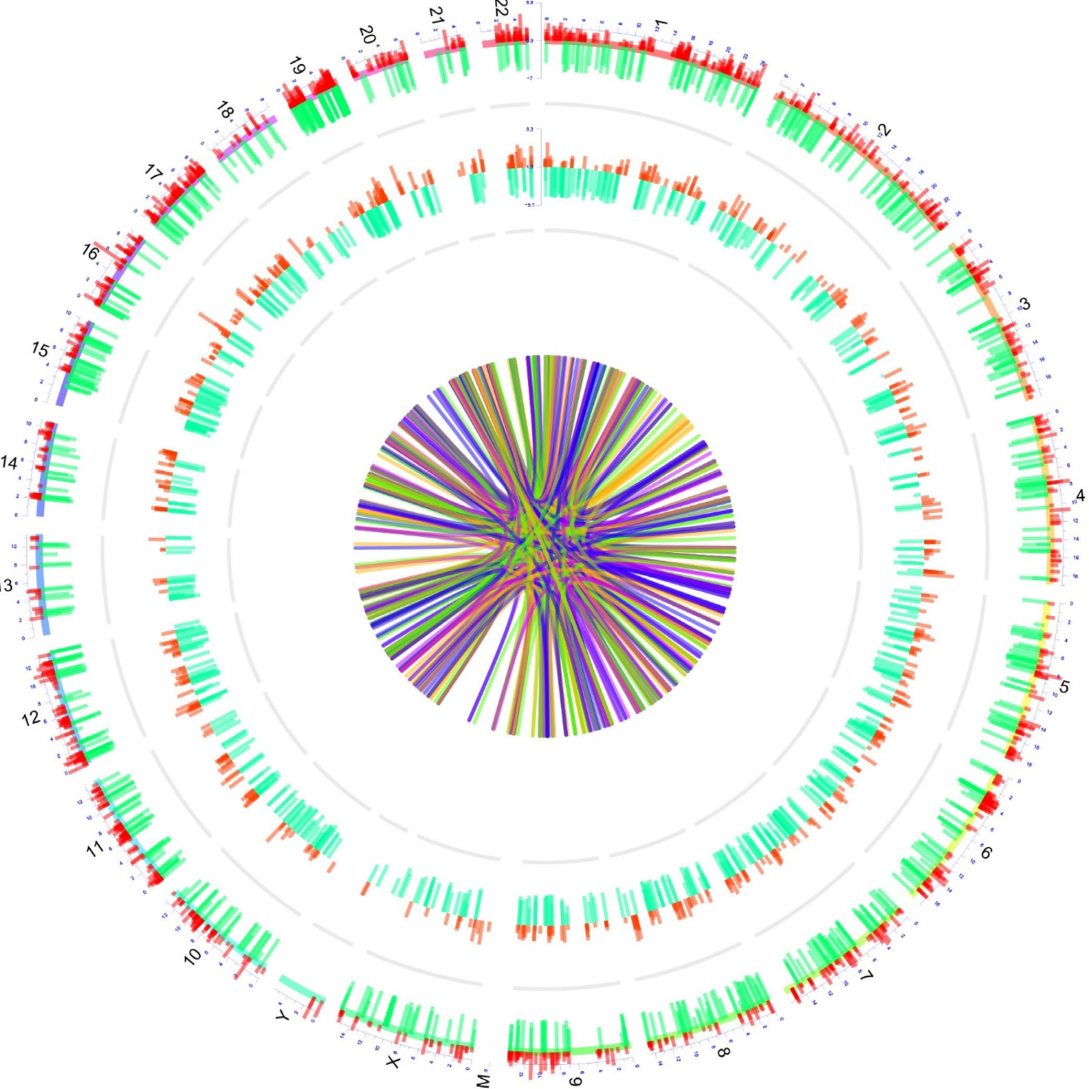
Transcription and regulation information. The Circos graph for transcription and regulation information of lncRNAs, mRNAs, and lncRNA‐mRNA regulation information. lncRNA, long noncoding RNA

Top 20 upregulated lncRNAs and top 20 downregulated lncRNAs were selected based on the rank of the absolute value of Log_2_FC, which are presented in Table 3. Afterward, the regulatory network of the trans‐ and cis‐regulations of these dysregulated lncRNAs with mRNAs is presented in Figure 4. Then, the Circos graph for transcription and regulation information disclosed the expression pattern of dysregulated mRNAs in different chromosomes in the first cycle, the expression pattern of dysregulated lncRNAs in distinctive chromosomes in the second circle, and the regulatory network of dysregulated lncRNA‐mRNA interactions in the inner collected lines (Figure 5).

**Table 3:** Top 40 dysregulated lncRNAs (20 up and 20 down) in AML patients compared to controls

Gene symbol	Gene ID	Location	Log_2_FC	*P* value	Trend
LncRNA CES1P1	ENSG00000228695	Chromosome 16	8.27641	2.2E‐12	Up
LncRNA RP4‐576H24.2	ENSG00000242324	Chromosome 20	6.793759	5.18E‐07	Up
LncRNA LINC01262	ENSG00000250739	Chromosome 4	6.712888	2.95E‐07	Up
LncRNA SIGLEC16	ENSG00000161643	Chromosome 19	6.088551	6.19E‐07	Up
LncRNA OR7E140P	ENSG00000238152	Chromosome 12	5.888917	3.65E‐05	Up
LncRNA LINC00977	ENSG00000250400	Chromosome 8	5.778342	1.67E‐06	Up
LncRNA RP11‐452K12.7	ENSG00000231970	Chromosome 10	5.728152	1.58E‐05	Up
LncRNA RP11‐1082L8.4	ENSG00000255491	Chromosome 8	5.469015	2.8E‐08	Up
LncRNA RP5‐1092A11.5	ENSG00000227589	Chromosome 1	5.349272	.000152	Up
LncRNA TLR8‐AS1	ENSG00000233338	Chromosome X	5.255034	.000185	Up
LncRNA PLBD1‐AS1	ENSG00000256751	Chromosome 12	5.229375	.000118	Up
LncRNA AC008268.2	ENSG00000237510	Chromosome 2	5.118274	8.54E‐06	Up
LncRNA AATBC	ENSG00000215458	Chromosome 21	5.061783	9.78E‐12	Up
LncRNA Y_RNA‐1	ENSG00000202019	Chromosome 22	5.030848	.000172	Up
LncRNA RUVBL1‐AS1	ENSG00000239608	Chromosome 3	4.998685	.000288	Up
LncRNA AC023137.2	ENSG00000228950	Chromosome 2	4.971807	.0001	Up
LncRNA CTD‐2589M5.5	ENSG00000254639	Chromosome 11	4.925658	3.35E‐05	Up
LncRNA Y_RNA‐2	ENSG00000202272	Chromosome X	4.875756	.000387	Up
LncRNA MFSD1P1	ENSG00000261868	Chromosome 17	4.846542	.000405	Up
LncRNA LILRP2	ENSG00000170858	Chromosome 19	4.8373	.000667	Up
LncRNA DIO3OS	ENSG00000258498	Chromosome 14	−5.68275	.000431	Down
LncRNA MEG3	ENSG00000214548	Chromosome 14	−5.47706	9.54E‐05	Down
LncRNA ST3GAL6‐AS1	ENSG00000239445	Chromosome 3	−5.34883	1.68E‐05	Down
LncRNA OR51A10P	ENSG00000230484	Chromosome 11	−4.81509	.000509	Down
LncRNA RP5‐983L19.2	ENSG00000226954	Chromosome 22	−4.73941	9.27E‐05	Down
LncRNA SNORD116‐8	ENSG00000207093	Chromosome 15	−4.72339	.00055	Down
LncRNA RP4‐717I23.2	ENSG00000229567	Chromosome 1	−4.62583	.000174	Down
LncRNA RP11‐618I10.1	ENSG00000249763	Chromosome 4	−4.61012	.000505	Down
LncRNA RP11‐556N21.1	ENSG00000243008	Chromosome 13	−4.31828	.001538	Down
LncRNA RP11‐227G15.9	ENSG00000264083	Chromosome 17	−4.30979	.002633	Down
LncRNA RP11‐488L18.8	ENSG00000232347	Chromosome 1	−4.27859	.000379	Down
LncRNA RP11‐283G6.3	ENSG00000256894	Chromosome 12	−4.21832	.000813	Down
LncRNA LINC01222	ENSG00000233410	Chromosome 1	−4.1961	.002546	Down
LncRNA RP11‐467L19.8	ENSG00000258488	Chromosome 15	−4.11613	.00039	Down
LncRNA LIFR‐AS1	ENSG00000244968	Chromosome 5	−4.03826	.015015	Down
LncRNA TPTEP1	ENSG00000100181	Chromosome 22	−4.00778	.000686	Down
LncRNA RP11‐214K3.18	ENSG00000270095	Chromosome 12	−3.97757	.001971	Down
LncRNA PDCL3P6	ENSG00000224255	Chromosome 1	−3.97002	.003771	Down
LncRNA RP11‐43F13.3	ENSG00000215246	Chromosome 5	−3.94694	.017161	Down
LncRNA LINC01252	ENSG00000247157	Chromosome 12	−3.89722	5.17E‐06	Down

Note

Top 40 dysregulated (20 upregulated and 20 downregulated) lncRNAs were selected based on the rank of absolute value of Log_2_FC.

Abbreviations: AML, acute myeloid leukemia; FC, fold change; lncRNA, long noncoding RNA.

### 3.5 Clinical characteristics of AML patients in RT‐qPCR validation

Ten candidate lncRNA expressions in bone marrow including top 5 upregulated lncRNAs and top 5 downregulated lncRNAs by RNA sequencing were selected for RT‐qPCR validation in 110 AML patients and 40 controls. As for the AML patients included in RT‐qPCR validation, the mean age was 43.1 ± 13.4 years, with 63 (57.3%) males and 47 (42.7%) females (Table 2). In addition, there were, respectively, 3 (2.7%), 35 (31.8%), 31 (28.2%), 34 (30.9%), and 7 (6.4%) patients who were in FAB classification of M1, M2, M4, M5, and M6. And, the number of patients who had risk stratification of better‐risk, intermediate‐risk, and poor‐risk were 28 (25.5%), 45 (40.9%), and 37 (33.6%), respectively. Additionally, the mean WBC was 15.4 (6.9‐28.4) × 10^9^/L.

### 3.6 Ten candidate lncRNA expressions in AML patients

**Figure 6:**
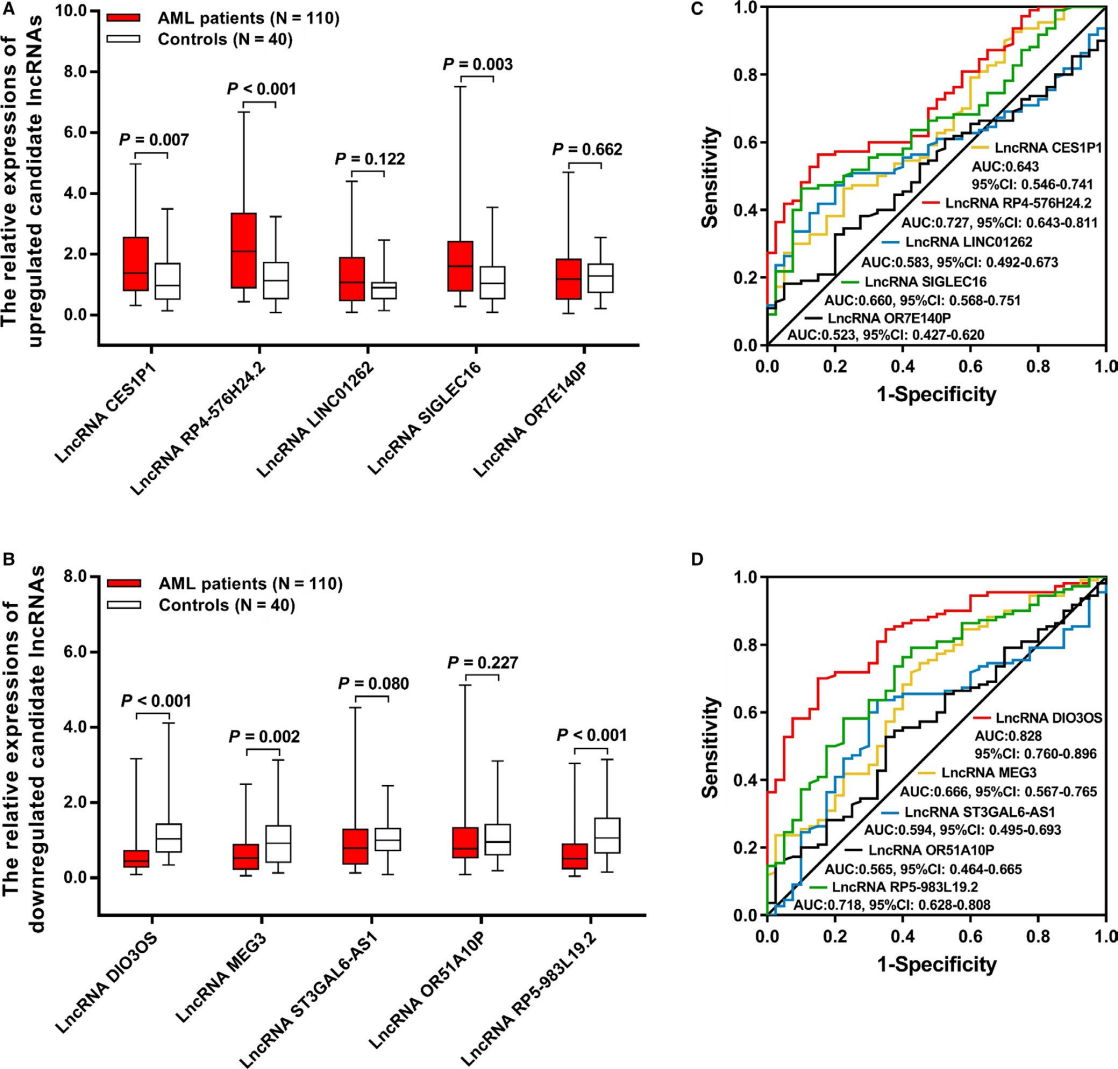
Expressions of 10 candidate lncRNAs. Expressions of upregulated lncRNAs (A), ROC curves of upregulated lncRNAs in predicting AML risk (B), expressions of downregulated lncRNAs (C), and ROC curves of downregulated lncRNAs in predicting AML risk (D). Comparison between two groups was determined by Wilcoxon rank sum test. Predictive value of candidate parameters for disease risk was detected by ROC curve and assessed by AUC *P* value <.05 was considered significant. AML, acute myeloid leukemia; ROC, receiver operating characteristic; AUC, area under curve; lncRNA, long noncoding RNA

For the top 5 upregulated candidate lncRNAs, the expressions of lncRNA CES1P1 (*P* = .007), lncRNA RP4‐576H24.2 (*P* < .001), and lncRNA SIGLEC16 (*P* = .003) were elevated in AML patients compared with those in controls; however, no difference of lncRNA LINC01262 (*P* = .122) or lncRNA OR7E140P (*P* = .662) expressions was found between AML patients and controls (Figure 6A). Then, the ROC curve analysis showed that lncRNA LINC01262 and lncRNA OR7E140P have no ability in differentiating AML patients from controls, while lncRNA CES1P1, lncRNA RP4‐576H24.2, and lncRNA OR7E140P distinguished AML patients from controls with relatively good AUC values, and lncRNA RP4‐576H24.2 presented with the highest AUC value of 0.727 (95% CI: 0.643‐0.811) (Figure 6C). With respect to the top 5 downregulated candidate lncRNAs, lncRNA DIO3OS (*P* < .001), LncRNA MEG3 (*P* = .002), and lncRNA RP5‐983L19.2 (*P* < .001) were downregulated in AML patients compared with controls, while the expressions of lncRNA ST3GAL6‐AS1 (*P* = .080) and lncRNA OR51A10P (*P* = .227) were similar between AML patients and controls (Figure 6B). And, ROC curve analysis disclosed that lncRNA ST3GAL6‐AS1 as well as lncRNA OR51A10P could not separate AML patients from controls, while lncRNA DIO3OS, lncRNA MEG3, and lncRNA PR5‐983L19.2 had good values in differentiating AML patients from controls, in which lncRNA DIO3OS had the finest AUC value of 0.828 (95% CI: 0.760‐0.896) (Figure 6D).

### 3.7 Correlations of 10 candidate lncRNA expressions with AML risk stratification

Only lncRNA RP4‐576H24.2 expression in bone marrow was correlated with AML risk stratification, to be exact, the level of lncRNA RP4‐576H24.2 was the highest in patients with poor‐risk followed by patients with intermediate‐risk, and was the lowest in patients with better‐risk (*P* = .009) (Table 4). In addition, the expressions of lncRNA CES1P1 (*P* = .074), lncRNA SIGLEC16 (*P* = .924), lncRNA DIO3OS (*P* = .445), lncRNA MEG3 (*P* = .158), lncRNA ST3GAL6‐AS1 (*P* = .171), lncRNA OR51A10P (*P* = .283), or lncRNA RP5‐983L19.2 (*P* = .109) were of no difference among patients at better‐risk, intermediate‐risk, and poor‐risk.

**Table 4:** Correlation of eight candidate lncRNAs with risk stratification of AML

Parameters	Risk stratification			*P* value
	Better‐risk (n = 28)	Intermediate‐risk (n = 45)	Poor‐risk (n = 37)	
LncRNA, median (IQR)				
LncRNA CES1P1	0.920 (0.717‐2.168)	1.918 (0.926‐2.792)	1.309 (0.755‐2.023)	.074
LncRNA RP4‐576H24.2	1.313 (0.664‐2.760)	1.581 (1.046‐2.653)	2.885 (1.547‐4.220)	.009
LncRNA SIGLEC16	1.570 (1.062‐2.244)	1.514 (0.745‐2.314)	1.849 (0.731‐2.757)	.924
LncRNA DIO3OS	0.590 (0.281‐0.840)	0.429 (0.258‐0.643)	0.437 (0.304‐0.730)	.445
LncRNA MEG3	0.644 (0.349‐1.079)	0.496 (0.198‐0.790)	0.402 (0.179‐0.979)	.158
LncRNA ST3GAL6‐AS1	0.922 (0.521‐1.963)	0.721 (0.341‐1.240)	0.726 (0.299‐1.101)	.171
LncRNA OR51A10P	0.732 (0.398‐1.285)	0.717 (0.514‐1.287)	0.878 (0.647‐1.392)	.283
LncRNA RP5‐983L19.2	0.371 (0.140‐0.777)	0.688 (0.329‐1.028)	0.443 (0.266‐0.980)	.109

Note

Difference was determined by Kruskal‐Wallis H test.

Abbreviations: AML, acute myeloid leukemia; IQR, interquartile range.

### 3.8 Correlations of 10 candidate lncRNA expressions with EFS and OS in AML patients

**Figure 7:**
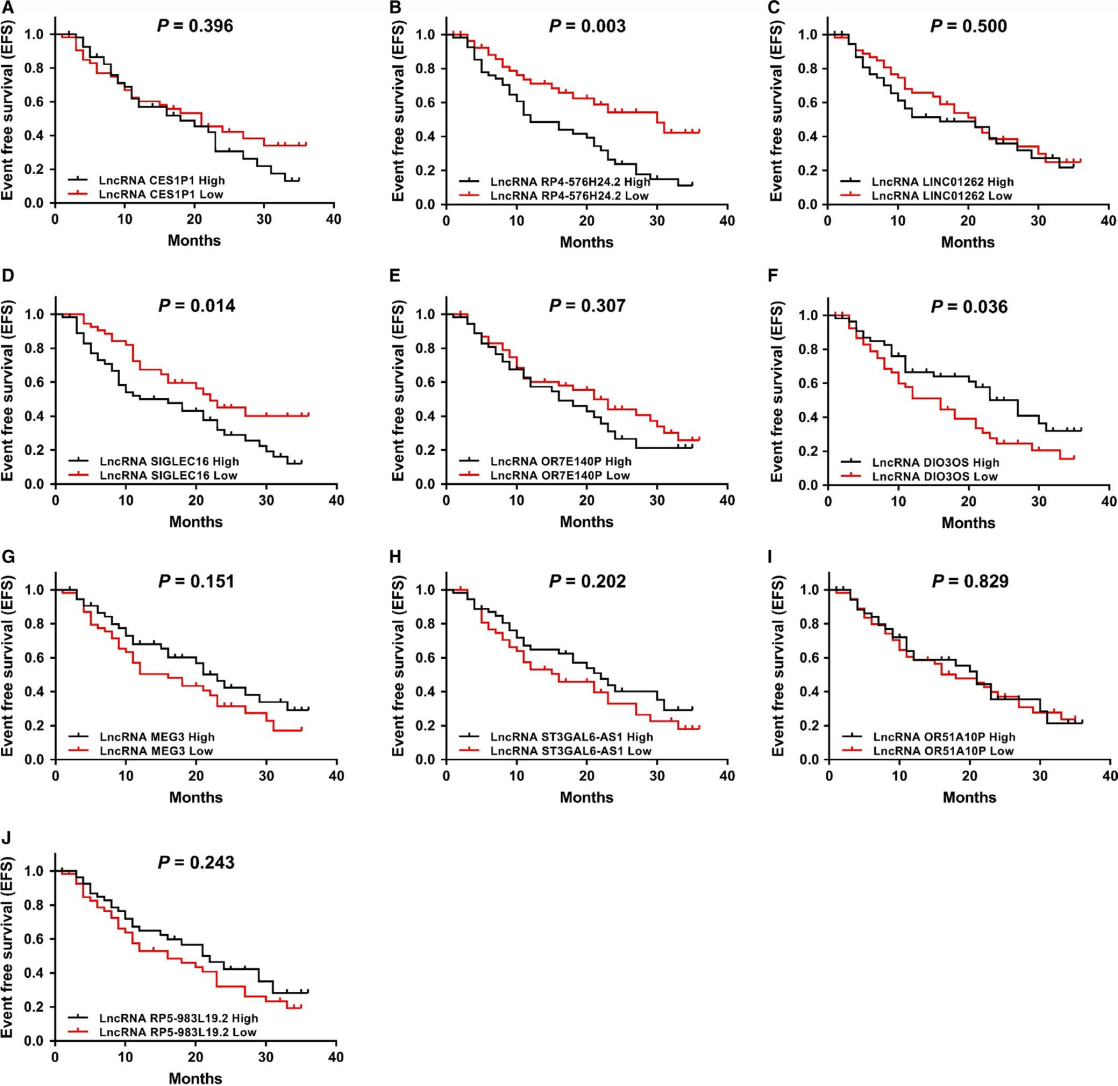
Correlations of 10 candidate lncRNAs with EFS in AML patients. The correlations of lncRNA CES1P1 (A), lncRNA RP4‐576H24.2 (B), lncRNA LINC01262 (C), lncRNA SIGLEC16 (D), lncRNA OR7E140P (E), lncRNA DIO3OS (F), lncRNA MEG3 (G), lncRNA ST3GAL6‐AS1 (H), lncRNA OR51A10P (I), and lncRNA RP5‐983L19.2 (J) expressions with EFS in 110 AML patients. Comparison of EFS was detected by Kaplan‐Meier curve and log‐rank test. *P* value <.05 was considered as significant. EFS, event‐free survival; AML, acute myeloid leukemia; lncRNA, long noncoding RNA

**Figure 8:**
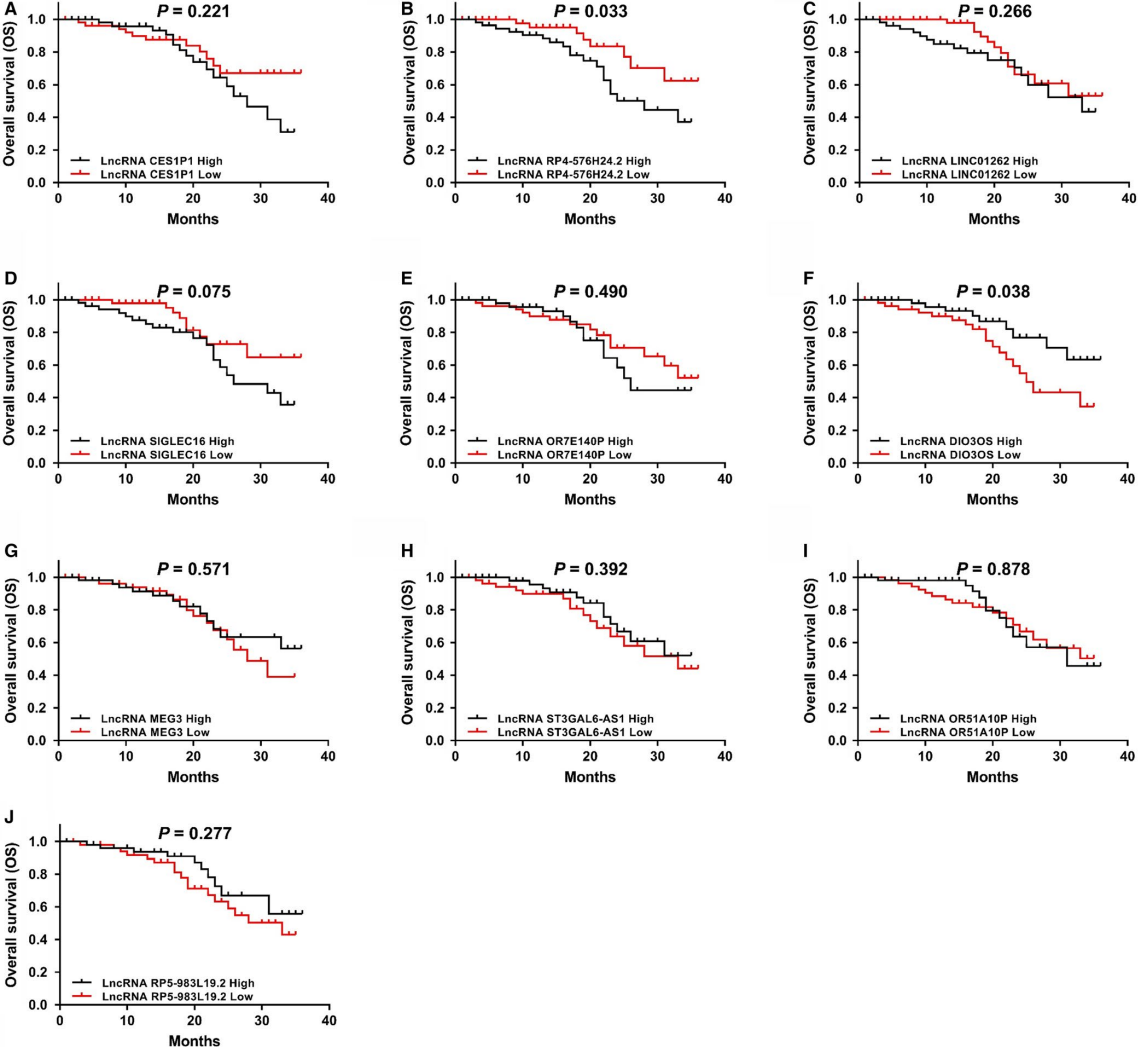
Correlations of 10 candidate lncRNAs with OS in 110 AML patients. The associations of lncRNA CES1P1 (A), lncRNA RP4‐576H24.2 (B), lncRNA LINC01262 (C), lncRNA SIGLEC16 (D), lncRNA OR7E140P (E), lncRNA DIO3OS (F), lncRNA MEG3 (G), lncRNA ST3GAL6‐AS1 (H), lncRNA OR51A10P (I), and lncRNA RP5‐983L19.2 (J) expressions with OS in AML patients. Comparison of OS was detected by Kaplan‐Meier curve and log‐rank test. *P* value <.05 was considered as significant. OS, overall survival; AML, acute myeloid leukemia; lncRNA, long noncoding RNA

LncRNA RP4‐576H24.2 (*P* = .003) (Figure 7B) high expression, lncRNA SIGLEC16 (*P* = .014) (Figure 7D) high expression, and lncRNA DIO3OS (*P* = .036) (Figure 7F) low expression were correlated with worse EFS, while no correlation was found between the other candidate lncRNAs with EFS (all *P* > .05) (Figure 7A,C,E,G‐J) in AML patients. As for OS, increased lncRNA RP4‐576H24.2 level (*P* = .033) (Figure 8B) and decreased lncRNA DIO3OS level (*P* = .038) (Figure 8F) were associated with declined OS, while the other candidate lncRNAs were not associated with OS (all *P* > .05) (Figure 8A,C‐E,G‐J). Then, due to that lncRNA RP4‐576H24.2 level was elevated in AML patients and had a good value in predicting AML risk, and an increased lncRNA RP4‐576H24.2 was correlated with poorer risk stratification and decreased EFS as well as OS, indicating that lncRNA RP4‐576H24.2 might be closely implicated in AML pathogenesis. Thus, we chose lncRNA RP4‐576H24.2 for the following in vitro experiments.

### 3.9 Comparison of candidate lncRNA expressions among patients with different FAB classifications

No difference of LncRNA CES1P1 (*P* = .473), LncRNA RP4‐576H24.2 (*P* = .640), LncRNA LINC01262 (*P* = .802), LncRNA SIGLEC16 (*P* = .656), LncRNA OR7E140P (*P* = .178), LncRNA DIO3OS (*P* = .602), LncRNA MEG3 (*P* = .792), LncRNA ST3GAL6‐AS1 (*P* = .269), LncRNA OR51A10P (*P* = .334), or LncRNA RP5‐983L19.2 (*P* = .235) expressions among patients with different FAB classifications was found (Table 5). These results indicated that the FAB classification did not affect the expressions of candidate lncRNAs in AML patients.

**Table 5:** Correlation of 10 candidate lncRNAs with FAB classification of AML

Parameters	FAB classification^a^					*P* value
	M1^a^ (n = 3)	M2 (n = 35)	M4 (n = 31)	M5 (n = 34)	M6 (n = 7)	
LncRNAs, median (IQR)						
LncRNA CES1P1	3.877 (0.590‐)	1.230 (0.700‐2.289)	1.748 (0.887‐2.264)	1.453(0.806‐2.369)	2.580 (1.088‐3.213)	.473
LncRNA RP4‐576H24.2	1.944 (0.901‐)	2.298 (0.878‐3.470)	1.495 (0.781‐3.120)	2.508 (1.033‐4.050)	2.412 (1.581‐4.642)	.640
LncRNA LINC01262	0.460 (0.129‐)	0.989 (0.482‐1.879)	1.125 (0.451‐1.918)	0.937 (0.506‐1.899)	1.561 (0.471‐2.203)	.802
LncRNA SIGLEC16	1.984 (1.664‐)	1.623 (0.741‐2.568)	1.610 (0.869‐2.303)	1.620 (0.660‐2.523)	1.178 (0.485‐1.555)	.656
LncRNA OR7E140P	2.216 (2.041‐)	1.079 (0.532‐1.746)	1.064 (0.367‐2.171)	1.120 (0.469‐1.498)	1.621 (1.305‐2.039)	.178
LncRNA DIO3OS	0.431 (0.346‐)	0.580 (0.320‐0.785)	0.429 (0.185‐0.736)	0.441 (0.269‐0.716)	0.346 (0.303‐0.758)	.602
LncRNA MEG3	0.792 (0.199‐)	0.530 (0.309‐1.045)	0.472 (0.197‐0.787)	0.551 (0.274‐0.838)	0.402 (0.195‐0.812)	.792
LncRNA ST3GAL6‐AS1	0.534 (0.133‐)	0.592 (0.321‐1.113)	0.799 (0.336‐1.197)	0.842 (0.474‐1.589)	0.869 (0.721‐1.103)	.269
LncRNA OR51A10P	1.043 (0.866‐)	0.757 (0.363‐1.129)	0.847 (0.628‐1.341)	0.816 (0.576‐1.559)	0.533 (0.394‐0.717)	.334
LncRNA RP5‐983L19.2	0.907 (0.336‐)	0.383 (0.213‐0.770)	0.722 (0.339‐1.108)	0.448 (0.232‐0.927)	0.602 (0.141‐0.782)	.235

Note

Difference was determined by Kruskal‐Wallis H test.

Abbreviations: AML, acute myeloid leukemia; FAB classification, French‐American‐Britain classification systems; IQR, interquartile range.

a Due to less patients in M1, the third quartile of lncRNAs expression cannot be calculated.

### 3.10 Effect of lncRNA RP4‐576H24.2 on AML cell proliferation and apoptosis

**Figure 9:**
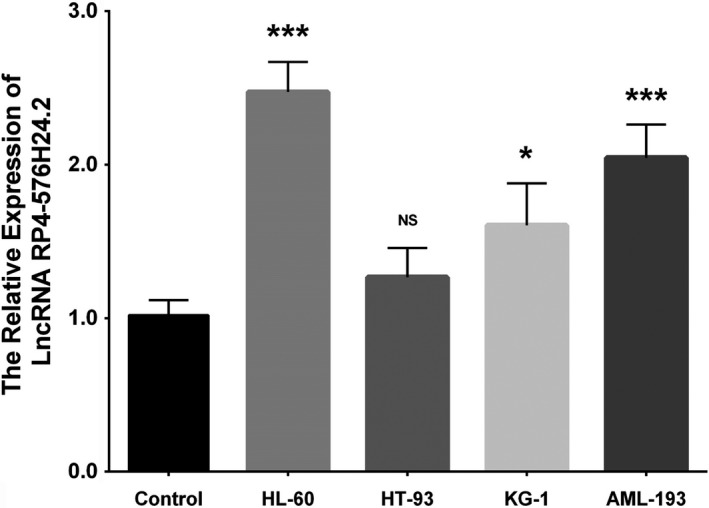
Expression of lncRNA RP4‐576H24.2 in AML cell lines. The lncRNA RP4‐576H24.2 expression in control, HL‐60, HT‐93, KG‐1, and AML‐193 cell lines. Comparison among multiple groups was detected by one‐way ANOVA followed by multiple comparison test. *P* value <.05 was considered as significant. *P value <.05; ***P value <.001. AML, acute myeloid leukemia; lncRNA, long noncoding RNA; ANOVA, one‐way analysis of variance

**Figure 10:**
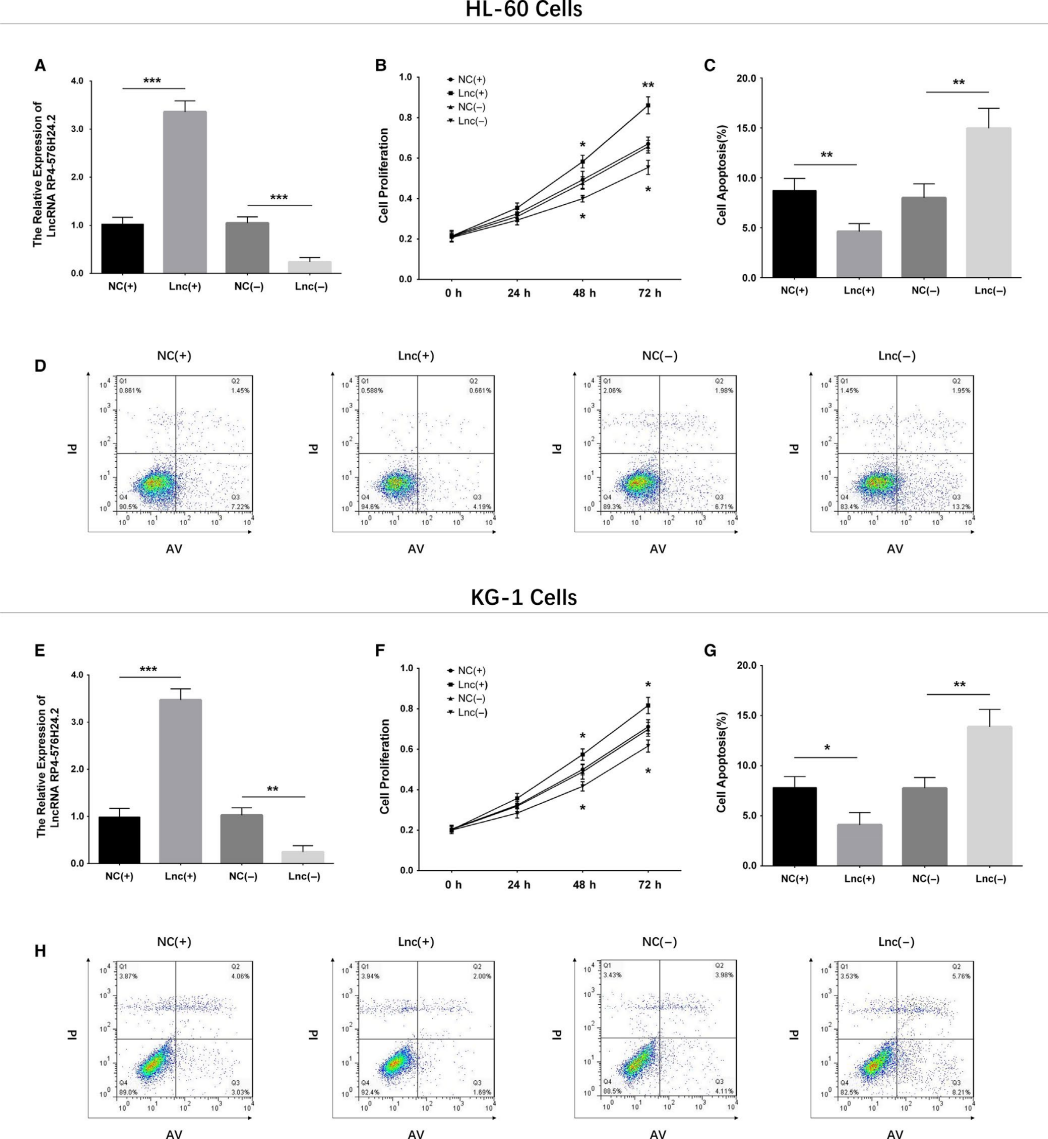
Effect of lncRNA RP4‐576H24.2 on proliferation and apoptosis in two AML cell lines. Expression of lncRNA RP4‐576H24.2 after transcriptions (A), and the effect of lncRNA RP4‐576H24.2 on cell proliferation (B) as well as cell apoptosis (C, D) in HL‐60 cells. The lncRNA RP4‐576H24.2 level after transcriptions (E), effect of lncRNA RP4‐576H24.2 on cell proliferation (F), and apoptosis (G, H) in KG‐1 cells. Comparison between two groups was determined by *t* test. *P* value <.05 was considered as significant. *P value <.05; **P value <.01; ***P value <.001. AML, acute myeloid leukemia; lncRNA, long noncoding RNA

LncRNA RP4‐576H24.2 expression was increased in HL‐60 cells (*P* < .001), KG‐1 cells (*P* < .05), and AML‐193 cells (*P* < .001), while was of no difference in HT‐93 cells (*P* > .05) compared with control cells (Figure 9). Then, the effect of lncRNA RP4‐576H24.2 on AML cell functions was evaluated in HL‐60 cell line and KG‐1 cell line. In HL‐60 cells and KG‐1 cells, the expression of lncRNA RP4‐576H24.2 was elevated in Lnc(+) group compared with NC(+) group (*P* < .001, *P* < .001), and was downregulated in Lnc(−) group compared with NC(−) group (*P* < .001, *P* < .001) (Figure 10A,E), indicating the transfections were successful. And, the cell proliferation was enhanced in Lnc(+) group than that in NC(+) group at 48 h (*P* < .05, *P* < .05) and 72 hours (*P* < .01, *P* < .05) while was repressed in Lnc(−) group compared with NC(−) group at 48 hours (*P* < .05, *P* < .05) and 72 hours (*P* < .01, *P* < .05) (Figure 10B,F) in both HL‐60 cells and KG‐1 cells. As for cell apoptosis, it was downregulated in Lnc(+) group compared with NC(+) group (*P* < .01, *P* < .05), while was upregulated in Lnc(−) group than that in NC(−) group (*P* < .01, *P* < .01) at 24 hours after transfections in HL‐60 cells and KG‐1 cells (Figure 10C,D,G,H). These results suggested that lncRNA RP4‐576H24.2 promoted cell proliferation while suppressed cell apoptosis in AML.

## 4 DISCUSSION

Profiting from the development of RNA sequencing and bioinformatics analysis, investigation of the heterogenetic pathology of AML has become more profound, among which the role of ncRNAs in development and progression of AML has been increasingly revealed in recent decades, including forming regulatory networks that could mediate multiple pathways related to AML.^15^, ^16^, ^17^ Nonetheless, previous studies investigating the roles of lncRNAs in AML only begin recently, and are quite limited, even though lncRNAs have been found to be crucial genetic factors in other malignancies.^18^, ^19^

In terms of the studies including RNA sequencing in AML, in a study using miRNA sequencing and transcription factor (TF) activity array, 308 dysregulated miRNAs and 84 dysregulated TFs are detected, then 1462 miRNA‐target gene pairs, 982 TF‐target gene pairs, and 196 TF‐miRNA pairs are identified subsequently; after emerging as a regulatory network of these dysregulated miRNAs and TF, the KEGG pathway analysis finds that the network is markedly enriched in 33 pathways with the AML‐related pathways the most significant.^20^ Another previous study performs whole‐genome microarrays in extramedullary infiltration (EMI) AML patients and non‐EMI AML patients, and discloses that 253 circular RNAs (circRNAs) and 663 mRNAs are upregulated, but 259 circRNAs and 838 mRNAs are downregulated in EMI AML patients compared to non‐EMI AML patients, then further enrichment analysis finds that these dysregulated circRNAs and mRNAs are mainly enriched in cell adhesion, migration, signal transduction, and cell to cell communications.^21^ With respect to lncRNA, there are also studies evaluating its expression pattern in AML. However, these studies are very few. A previous study using microarray and bioinformatics analyses reveals that in pediatric AML patients, 372 lncRNAs and 136 mRNAs are found to be dysregulated in patients compared with normal controls, and further RT‐qPCR validates that the most dysregulated lncRNAs in pediatric AML patients is lnc RNA ENST00000435695.^22^ And, another study assessing the lncRNA expression profile in cytogenetically normal AML patients observes a specific lncRNA expression profile which is dependent on the mutational status of NPM1 gene, and finds 12 lncRNAs being able to distinguish NPM1‐mutated patients from NPM1 wild‐type patients; in addition, they also discover that lncRNA XLOC_109948 is associated with drug resistance and prognosis.^23^ However, these previous studies which investigate lncRNA expression pattern in AML are performed in different patient cohorts, such as the pediatric AML patients (vs healthy children) and cytogenetically normal AML patients (vs cytogenetically abnormal AML patients). And the study focusing on the implication of lncRNA expression pattern in adult AML patients (vs adult controls) is not reported yet. In this study, we found that the lncRNA expression pattern could differentiate adult AML patients from controls, then 630 upregulated lncRNAs and 621 downregulated lncRNAs in AML patients were identified, and further enrichment analysis showed that these dysregulated lncRNAs were enriched in AML pathology‐related biological processes (such as neutrophil degranulation, inflammatory response as well as immune response), and AML‐related signaling pathways (including leukocyte transendothelial migration, hematopoietic cell lineage, apoptosis and so on), and were predominantly located in cell lysosomes and extracellular exosomes. Our results indicated that these dysregulated lncRNAs might act as crucial players in AML pathology mainly through mediating neutrophil degranulation and pathway of leukocyte transendothelial migration in cell lysosomes and extracellular exosomes.

The poor prognosis of AML has highlighted the need for searching more novel and reliable biomarkers which can assist in the management of AML patients, and several specific lncRNAs have been proposed to exhibit prognostic value. For instance, the lncRNA LINC00265 is reported to be upregulated in bone marrow in AML patients than that in controls and is associated with worse OS in AML patients.^24^ Another recent study elucidates that higher levels of lncRNA CCAT1 and lncRNA PVT1 in peripheral blood mononuclear cells are associated with poor prognosis in AML patients.^25^ In addition, a recent study reports that lncRNA in nonhomologous end joining (NHEJ) pathway 1 (LINP1) is upregulated in pediatric and adolescent AML patients, and promotes the malignant behaviors in AML cells via regulating the HNF4α/AMPK/WNT5A signaling pathway.^26^ Another study illuminates that lncRNA HOXB‐AS3 enhances AML cell proliferation and its elevated expression predicts worse prognosis patients with AML and myelodysplastic syndrome.^27^ While the systemic investigation of multiple lncRNAs as biomarkers for AML risk and prognosis is not conducted yet. In our study, 10 candidate lncRNAs that were selected from RNA sequencing results were further validated by RT‐qPCR in 110 AML patients and 40 controls, and their correlations with disease risk and prognosis were assessed, which elucidated that there were six candidate lncRNAs correlated with AML risk, one candidate lncRNA associated with risk stratification, and three candidate lncRNAs correlated with patients’ survivals, in which lncRNA RP4‐576H24.2 was the only lncRNA which predicted AML risk, correlated with risk stratification, EFS, and OS, suggesting that lncRNA RP4‐576H24.2 might serve as a potential biomarker for diagnosis and prognosis in AML patients. However, lncRNA RP4‐576H24.2 was a lncRNA firstly reported in this study, no specific mechanism of this lncRNA in regulating AML pathogenesis could be found in any previous study. As for the possible explanations to our results, it might include: 1) lncRNA RP4‐576H24.2 may promote development and progression of AML through regulating multiple downstream genes as displayed in our RNA sequencing results, in which several genes regulated by lncRNA RP4‐576H24.2 are reported to participate in the pathogenesis of leukemia, such as PTPRJ, FCN1, KLF4, G0S2, and LILRB2.^28^, ^29^, ^30^, ^31^, ^32^ Therefore, lncRNA RP4‐576H24.2 might promote the development or progression of AML by mediating the leukemia pathology‐related genes, such as PTPRJ, FCN1, KLF4, G0S2, and LILRB2; 2) our further cell experiments disclosed that lncRNA RP4‐576H24.2 promoted proliferation while inhibited apoptosis of two AML cell lines, indicating that lncRNA RP4‐576H24.2 might aggravate the progression of AML through regulating AML cell functions.

In addition, there were several issues that need to be illustrated in this study. First, there were patients with FAB classification of M1 and M6 in the 110 AML patients in RT‐qPCR validation; however, no patients with FAB classification of M1 or M6 were included in the four AML patients in RNA sequencing. This heterogeneity might result from that the sample size was much larger in RT‐qPCR validation and all the patients were enrolled consecutively, thus, there were patients with more diverse FAB classifications in RT‐qPCR validation. However, we further analyzed the difference of candidate lncRNA expressions among patients with different FAB classifications, which disclosed that candidate lncRNA expressions did not vary among patients with different FAB classifications. These results indicated that the FAB classification was not a factor affecting the candidate lncRNA expressions in AML patients. Second, lncRNA LINC01262 and lncRNA OR7E140P expressions were markedly dysregulated in AML patients compared with controls in RNA sequencing, while did not show any difference between AML patients and controls in RT‐qPCR validation, which might derive from that: (a) the results in RNA sequencing might be affected by a singular value due to that the sample size for RNA sequencing was small; (b) the RNA sequencing technique was less accurate than RT‐qPCR, which might also contribute to this conflict.

Furthermore, the underlying mechanisms of several specific lncRNAs in AML pathogenesis are also reported previously. For instance, knockdown of lncRNA ZFAS1 represses the AML progression through mediating the miR‐150/Sp1 and miR‐150/Myb pathways.^33^ And lncRNA ANRIL enhances the malignant cell survival and cell glucose metabolism of AML by regulating the AdipoR1/AMPK/SIRT1 pathway.^13^ In addition, lncRNA UCA1 expression is increased in AML pediatric patients after adriamycin (ADR)‐based chemotherapy, and knockdown of lncRNA UCA1 inhibits chemoresistance to ADR in AML cells via mediating miR‐125a/hexokinase 2 pathway.^11^ And lncRNA TUG1 suppresses AML cell proliferation while promotes apoptosis through targeting aurora kinase A.^12^ Furthermore, a recent experiment reveals that lncRNA SNHG1 promotes cell proliferation through mediating miR‐488‐5p/NUP205 axis in AML.^34^ In this study, due to that lncRNA RP4‐576H24.2 was the only candidate lncRNA correlated with AML risk, risk stratification, and prognosis of AML, we further performed in vitro experiments to explore the effect of lncRNA RP4‐576H24.2 on AML cell functions, which showed that lncRNA RP4‐576H24.2 was upregulated in AML cell lines compared with normal BMMCs, and it promoted cell proliferation as well as repressed cell apoptosis in two AML cell lines (HL‐60 cells and KG‐1 cells), which elucidated the potential mechanisms of lncRNA RP4‐576H24.2 in regulating the AML progression. To the best knowledge of ours, the lncRNA RP4‐576H24.2 was reported in AML for the first time by our study, and the probable explanations to the results in our in vitro experiments might be due to that lncRNA RP4‐576H24.2 could promote progressive cell functions via targeting multiple mRNAs, which was also shown in the regulatory network of lncRNA‐mRNA in our results.

In conclusion, the lncRNA expression pattern is closely involved in the development and progression of AML, and several specific lncRNAs exhibit potential to be biomarkers for AML risk as well as prognosis. Besides, lncRNA RP4‐576H24.2 might be a potential oncogene in AML pathogenesis. These data would shed light on the potential role of lncRNA in AML pathology and the application of lncRNA as a novel biomarker for AML management.

## CONFLICT OF INTEREST

The authors declare that they have no competing interests.
